# Lacrimal duct Surgery: Different Techniques and Long-Term Postoperative Results

**DOI:** 10.1007/s12070-024-04670-3

**Published:** 2024-06-01

**Authors:** Maria Casasayas, Humbert Massegur-Solench, María Martel-Marín, Kataryna Alicja Kolanczak, Anna Holgado, Juan Ramón Gras-Cabrerizo

**Affiliations:** 1 Otorhinolaryngology Department, Hospital de la Santa Creu i Sant Pau, Universitat Autònoma de Barcelona, C/ Mas Casanovas, 90, Barcelona, 08041 Spain; 2 Otorhinolaryngology Department, Clínica Corachan, Universitat Autònoma de Barcelona, Barcelona, Spain; 3 Otorhinolaryngology Department, Centro Médico Teknon, Barcelona, Spain; 4 Otorhinolaryngology Department, Hospital del Mar, Universitat Autònoma de Barcelona- Universitat Pompeu Fabra, Barcelona, Spain

**Keywords:** Epiphora, Lacrimal duct obstruction, Conjunctivodacryorhinostomy, Dacryocystorhinostomy, Endoscopic surgery

## Abstract

Pre-saccal obstructions of the lacrimal duct can be solved with a conjunctivodacryocystorhinostomy while saccal or post-saccal obstructions are restored with an external, endoscopic or laser dacryocystorhinostomy (DCR). The aim of the present study is to review the indications for each technique and to compare the results achieved. Retrospective review of 335 patients in whom 440 surgeries of the lacrimal duct were performed, with at least 4 months of follow-up. Outcomes in terms of symptoms and endoscopic findings during follow-up were considered. Successful results understood as resolution of symptoms were observed in 85% of cases treated with endoscopic DCR and in 62% of cases after laser DCR (*p* < 0.001). Among patients with recurrence of epiphora after surgery, 32% showed a visible ostium during endoscopy. Sixty-eight conjunctivodacryocystorhinostomies were performed, 68% of which required a tube replacement due to obstruction or extrusion. The mean duration of the tubes replaced was 10 months (range 3 days – 95 months). Endoscopic DCR shows better success rates than laser DCR. A considerable percentage of failures after DCR surgery present a visible ostium on endoscopic examination. This fact should lead to reconsider the initial diagnosis, ruling out functional problems or canalicular obstructions.

## Introduction

Epiphora is a common condition in clinical practice, and it is mainly caused by an imbalance between tear production and drainage. The impaired drainage may be due to an obstruction at any point along the tear pathway or to a functional disorder.

Anatomy plays a relevant role, since the location of the obstruction will determine the type of surgery required. Pre-saccal obstructions are located in the lacrimal punctum or canaliculus and the standard procedure to treat them is a conjunctivodacryocystorhinostomy (CDR) [1], which consists in creating a fistula from the conjunctiva to the nasal cavity with the insertion of a permanent tube. On the other hand, saccal or post-saccal obstructions are located in the lacrimal sac or nasolacrimal duct and can be solved with a dacryocystorhinostomy, which consists in creating a passage from the lacrimal sac to the nasal cavity. It can be achieved by different techniques: external, endonasal endoscopic (E-DCR) or endocanalicular with diode laser (L-DCR) [2]. 

The objective of the present study is to review the indications for each technique and to compare the results obtained by E-DCR and L-DCR during more than 10 years of clinical experience.

## Methods

The Ethics Committee waived the need of ethics approval given the retrospective nature of the study. Verbal informed consent was provided to fill a prospective database on the surgeries performed on each patient.

We conducted the present study retrospectively, obtaining the information from a prospectively completed database since 2009. All patients undergoing lacrimal surgery for nasolacrimal obstruction between 2009 and 2021 were reviewed. Patients were treated at four different hospitals, by four surgeons. We only considered patients with more than 4 months of follow-up after surgery or after removal of the silicone stent in cases where a stent was placed. In 6 recurrent patients, the stent was left permanently or semi-permanently, therefore they were excluded from the analysis.

A total of 440 surgeries were performed in 335 patients with epiphora and/or dacryocystitis; 69 affected bilaterally (20.6%) and 266 unilaterally (79.4%). There was a predominance of female sex (73.4%).

We performed 258 E-DCR (58.6%) and 114 L-DCR (25.9%). The mean ages of each surgical group were 63 years [standard deviation (SD) 14.8], and 68 years (SD 12.4), respectively (*p* = 0.001). We counted 24 cases of secondary surgeries (6.5%). We performed 68 CDRs (15.5%), whose mean age was 65 years (SD 11.8 months).

As noted by Gras et al. in 2012 [2], the E-DCR technique is indicated in most patients, leaving L-DCR for patients who refuse general anesthesia or when it is contraindicated, as long as the surgical anatomic exposure is favorable. In our consideration, external DCR should be performed in patients who cannot undergo general anesthesia and the anatomic exposure is unfavorable for L-DCR. It should also be considered in case of lacrimal sac atrophy, if a lacrimal duct tumor is suspected or after facial trauma. External DCRs have not been considered in the analysis due to lack of follow-up data.

E-DCR was performed according to the modified version of the technique described by Massegur in 2004 [3]. L-DCR was performed under local anesthesia using a 980 nm diode laser [2]. 

CDR was performed under local anesthesia, creating a new lacrimal pathway from the conjunctival sac to the nasal cavity. In most cases, to avoid granuloma formation or distal obstruction of the tube, the anterior third of the middle turbinate was removed.

We defined success as the absence of epiphora and dacryocystitis after surgery. For the statistical analysis we used SPSS, 20.0 version. The chi-square test or Fisher’s exact tests, as appropriate, were used to compare qualitative variables. Student’s t test was used to compare quantitative variables. Non-parametric statistics were used if not normal distribution was observed. Recurrence was computed using the Kaplan-Meier method and compared using the Mantel-Haenszel test. Endpoints were calculated from the date of stent removal to recurrence.

## Results

### Dacryocystorhinostomy

Among all DCRs, 290 (78%) surgeries had achieved successful results at 4-month of follow-up. By group, 84.9% of cases treated with E-DCR (219/258) and 62.3% of cases in the L-DCR group (71/114) (*p* < 0.001).

**Fig. 1:**
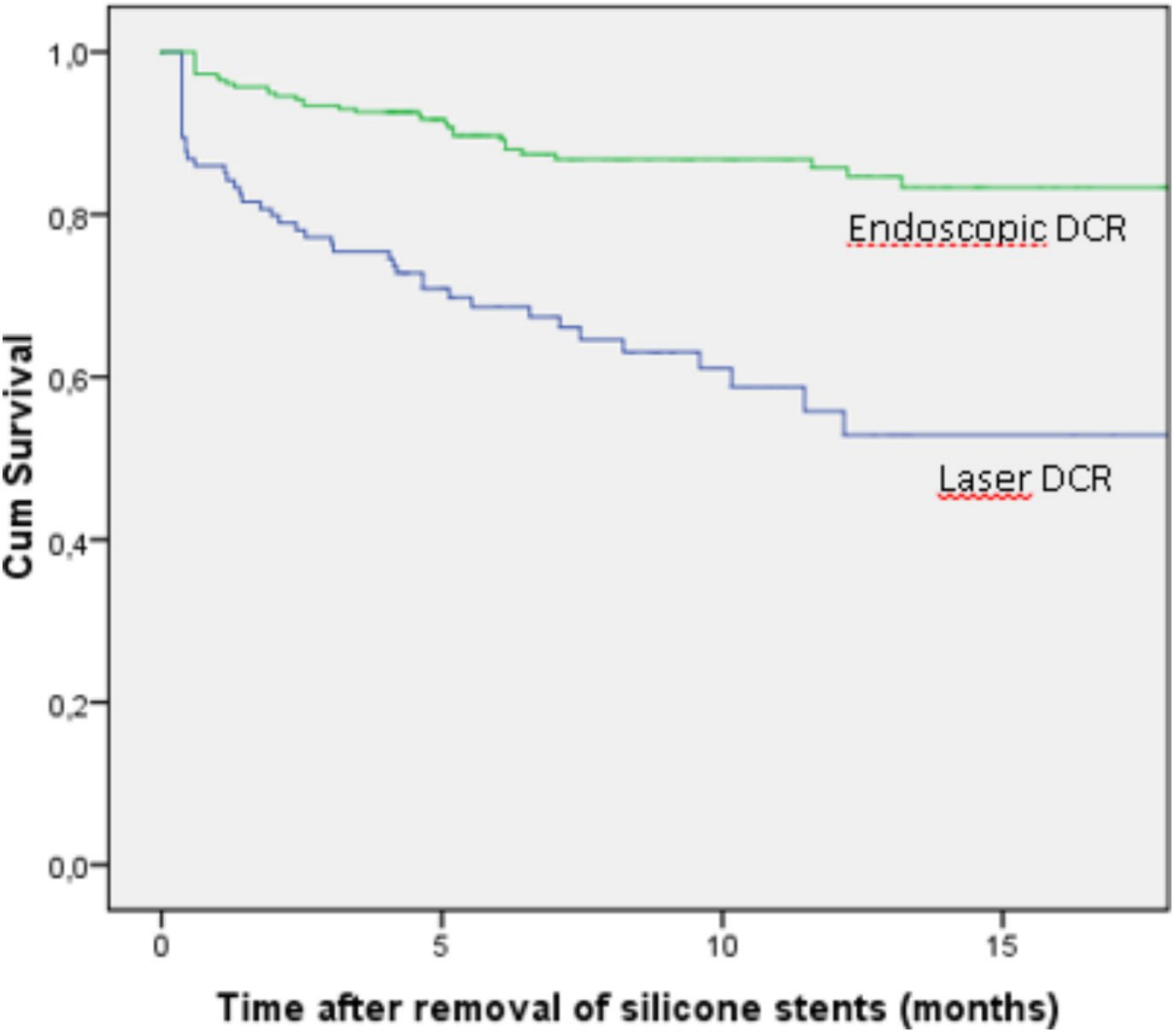
Kaplan-Mayer graph showing actuarial recurrence-free survival in months for E-DCR and L-DCR groups. Time is calculated after the removal of the silicone stent

Figure 1 shows a Kaplan-Meier plot reflecting actuarial recurrence-free survival in months for E-DCR and L-DCR groups. Recurrence-free survival at 12-months follow-up for E-DCR group was 84.6% (CI 95% 79.3–89.9) while that of L-DCR group was 52.9% (CI 95% 40.5–65.3). Among all DCRs, the median time between the silicone stent removal and reappearance of epiphora was 2.5 months (range 0–79). For the E-DCR group the median time to recurrence was 4.0 months, compared to 1.8 months for the L-DCR group (*p* = 0.003).

As can be seen in the Table 1, in most patients without postoperative epiphora the ostium was visible by endoscopic examination during follow-up (88%). On the contrary, as expected, in 68% of the patients with epiphora recurrence the ostium was not visible. In 12% of cases, although the ostium was not visible, the patient did not report epiphora and in 32% of the cases the patient had epiphora and the ostium was clearly seen.

**Table 1:** Endoscopic findings during follow-up according to the results of the surgery

	Success – No epiphora	Recurrence - Epiphora
Visible ostium	255 (88%)	26 (32%)
Non-visible ostium	35 (12%)	56 (68%)
Total	290	82

In most cases (100% of L-DCR and 90% of E-DCR) a bicanalicular silicone stent was placed to prevent fibrous closure of the ostium during the healing process. The stents where removed after a median of 1.9 months (range 0.4–16.8) in the E-DCR group and 2.4 months (range 0.5–13.9) in the L-DCR group (*p* < 0.001). Among patients with an E-DCR, the success rate when a stent was placed was 85% (198/233). Very similar to the rate in patients without a stent, which was 84% (21/25), (*p* = 1.000). Also among patients with an E-DCR, the median time to removal was comparable between successful and unsuccessful surgeries [1.9 months (range 0.5–16.8) vs. 2.0 months (range 0.3–5.1)]. Accidental stent extrusion occurred in 29 cases (19 E-DCR and 10 L-DCR). No differences were found between the percentage of epiphora recurrence between these patients and those with correct stent position [27.6% versus 22.0% recurrence, respectively (*p* = 0.643)].

No major complications were reported during the study period. Five orbital ecchymosis were described, 2 after L-DCR and 3 after E-DCR. Two episodes of epistaxis occurred after E-DCR and resolved after nasal packing. In the E-DCR group, 44 cases presented synechiae and in the L-DCR group, 19 cases. When analyzing the outcomes of these cases with synechiae, we observed that 30% (*n* = 19/63) had epiphora during follow-up compared to 20% (*n* = 63/309) among patients without synechiae (*p* = 0.088). Granulomas were found in 26 cases of E-DCR and 3 cases of L-DCR. Again, the results showed no differences between patients with and without granulomas (31% versus 21% of epiphora, respectively, *p* = 0.224).

### Conjunctivodacryocystorhinostomy (CDR)

A total of 68 CDRs were performed, placing 43 Metaireau tubes (63%) and 25 Lester Jones tubes (37%). We started placing Lester Jones tubes in 2018, prior to that time, only Metaireau tubes were available. Considering that this technique relies on the insertion of a prosthesis, we evaluated success by measuring the time until a replacement was required. Of the 68 tubes placed, 45 (68%) had to be removed due to extrusion, obstruction or recurrent conjunctivitis. In most cases, a new tube was placed. The median duration of the replaced tubes was 10 months (range 3 days − 96 months). However, some patients have not yet required replacement and continue to show long-term permeability of the tube (median 26 months, range 4–79 months).

No severe complications were observed after placement of an LCR tube. Five patients presented an orbital ecchymosis, 9 cases a granuloma and 4 cases postoperative synechiae.

## Discussion

Lacrimal duct obstructions are a frequent cause of epiphora. Before surgery, the keys to success are determining the cause of the epiphora, ruling out a functional disorder, and correctly locating the site of obstruction. During surgery, one of the most important factors is the size of the ostium (widest bony aperture) after opening the lacrimal sac [4, 5]. 

Dacryocystorhinostomy can be performed by external, endonasal or laser approaches with their several modifications. All three techniques usually show good results, with functional success rates above 80% in most publications [6–8]. For example, Balikoglu et al. [9] observed functional success rates of 81%, 72% and 73% for patients with external DCR, E-DCR and L-DCR respectively. Published results in E-DCR range from 80 to 97% [6]. Our results using the endonasal endoscopic approach are consistent with the literature, as we observed an 85% success rate in patients with at least 4-months follow-up.

The outcomes of the endonasal transcanalicular laser technique are controversial and differ greatly between studies, with rates ranging from 60 to 94% [8–10]. Our results after L-DCR are relatively low compared to the literature, with a success rate of 62%, maybe because some groups complement laser surgery with mechanical ostium enlargement procedures [11]. 

It is difficult to compare the outcomes with those of other groups because there is no standardized definition of success in lacrimal duct surgery. We considered that surgery was successful if the patient did not present epiphora during follow-up. Other groups defined it as an improvement of epiphora, the absence of new episodes of dacryocystitis and/or a patent ostium in the nasal cavity (anatomical success) [9, 12]. We consider that if the patient still refers epiphora after surgery, even if it has decreased, it should be considered as a recurrence or persistence.

Another possible explanation for our results is shown in Table 1, which compares endoscopic findings with epiphora during follow-up. When analyzed, we observed two presumable situations: the cases without epiphora that showed a visible ostium and the cases with epiphora in which the ostium had closed. However, we found it interesting to evaluate the rest of the cases. On the one hand, there were 12% of patients without postoperative epiphora in whom the ostium was not visible. This can be explained by the presence of synechiae or a membrane occluding the ostium, without altering its functionality. On the other hand, there were 32% of patients with persistent epiphora but with a visible ostium during endoscopy. Although there was an apparently satisfactory anatomical outcome, we consider these cases as recurrences, given the functional failure [4]. These cases present a challenge to the committee, as they may have a functional disorder already present before the surgery or perhaps a misdiagnosis was made. Konuk et al. [13] and lately Ekin et al. [5] observed that the most common causes of unsuccessful DCR surgery were inadequate size and location of the bony ostium, fibrosis at rhinostomy site, and canalicular obstruction. The latest represent a misdiagnosis of pre-saccal obstructions. Therefore, physicians should properly examine patients and consider the possibility of canalicular obstruction. In case of doubt, dacryocystography is a useful tool [14]. In 25% of our cases, a digital subtraction dacryocystography helped the committee to indicate surgery. Recently, computed tomography dacryocystography has been introduced as a complementary tool to document the anatomy of the lacrimal system and adjacent structures. Its use in clinical practice lies in complex obstructions after craniofacial trauma, congenital deformities, or neoplasms [14]. 

The time to recurrence was 2.5 months after the removal of the silicone stent. Golan et al. [15] have published that E-DCRs results after 2 weeks correlate with those observed after 6 months. Our results disagree with this observation, as many patients presented recurrence months after surgery. Bertaux et al. [16] described that the ostium of an E-DCR shrinks significantly within the 2 months after removal of the stent. According to Maini et al. [17], the success rate of L-DCR after 3 months of follow-up was 82%, compared to 68% after 12 months. They did not observe these differences in the E-DCR group, which maintained rates of 76% and 74%, respectively. In our sample, the success rate also remained stable at 12-month follow-up in the E-DCR group (85%) and, similar to Maini’s findings, in the L-DCR group it decreased from 62 to 53% after a year. In laser surgery, even lower functional success rates at 2 years follow-up have been described, 31% versus 88% observed at 3-months follow-up [11]. 

The use of silicone stents remains controversial. In 2011, a meta-analysis by Feng et al. [18] showed no statistical difference in the success rate between the DCR with and without stents. However, two recent meta-analysis support the opposite view [19, 20]. Both studies analyzed randomized controlled trials and observed better success rates in the stent group after external DCR. No statistically significant results were observed in the E-DCR group, but there was a trend towards the benefit of using stents. Most surgeons participating in this study continued to place stents after DCR surgery, but they have progressively reduced the time before its removal, especially in E-DCR. Our results did not yet show this change, given the accumulative data. However, they revealed that patients with or without stents showed very similar results.

Complications occasionally occurred after DCR surgery. Synechiae and granulomas can be considered as an unexpected event, not as a complication. Our results support this assumption, given that outcomes were comparable between patients with and without synechiae and granulomas. However, other studies showed worse results in patients that presented granulomas or synechiae [9]. 

Pre-saccal obstructions should be treated with a CDR and a tube placement. However, some authors such as Nomura et al. [7] performed E-DCR with stent insertion in these cases. They argued that the insertion of a stent is necessary in patients with canalicular stenosis because DCR alone does not restore canalicular patency. It is in this condition that they obtained worse results compared to other cases of DCR. Therefore, it reinforces the opinion that the communication created between the conjunctival sac and the nasal cavity in CDRs is the best treatment for these pre-saccal obstructions.

Lester Jones Tubes were first described in 1962 [21]. They are made of Pyrex glass and have been technically improved over the years. Metaireau tubes were described by Metaireau in 1979 and are made of polyvinylpyrrolidone (PVP) [22]. 

According to the literature, Lester Jones tubes are currently the most widely used. Bagdonaite et al. [1] studied the outcomes of Lester Jones tubes over a 12-year period and observed a success rate of 97%, defined as a patent and well-positioned tube. However, 43% of the tubes required replacement, and it was due to extrusion in half of the patients. The mean interval to tube replacement was between 7 and 12 months. [1] Even though the experience of the hospitals participating in this study is limited, our results are similar the those reported by Bagdonaite.

The advantage of Lester Jones tubes, being made of Pyrex glass, is that they get less clogged. However, this adds a major difficulty during replacement, as they can easily break. In our experience, this means that they cannot be removed in the consultation room, but have to be removed in the operating theatre under sedation.

E-DCR shows good success rates in patients with saccal or post-saccal obstruction of the lacrimal duct. Although outcomes after L-DCR are significantly worse, this surgery maintains its role in patients with contraindications to general anesthesia. A considerable percentage of failures after DCR surgery have a visible ostium on endoscopic examination. This fact should make us reconsider the initial diagnosis, ruling out functional problems or canalicular obstructions.
